# Poly[aqua­bis­(μ-formato-κ^2^ *O*:*O*′)(μ-pyrazine-κ^2^ *N*:*N*′)nickel(II)]

**DOI:** 10.1107/S1600536811011913

**Published:** 2011-04-07

**Authors:** Susanne Wöhlert, Mario Wriedt, Inke Jess, Christian Näther

**Affiliations:** a Institut für Anorganische Chemie, Christian-Albrechts-Universität Kiel, Max-Eyth-Str. 2, 24118 Kiel, Germany; b Departement of Chemistry, Texas A&M University, College Station, Texas 77843, USA

## Abstract

In the title compound, [Ni(CHO_2_)_2_(C_4_H_4_N_2_)(H_2_O)], the nickel(II) cations are coordinated by three *O*-bonded-formato anions, two *N*-bonded-pyrazine ligands and one water mol­ecule in an octa­hedral coordination mode. The nickel(II) cations are connected by *μ*-1,3-bridging formato anions and *N,N′*-bridging pyrazine ligands into a three dimensional coordination network. The asymmetric unit consists of one nickel(II) cation, one water mol­ecule and two crystallograph­ically independent formato anions in general positions as well as two crystallographically independent pyrazine ligands, which are located on centers of inversion.

## Related literature

For background of this work, see: Boeckmann & Näther (2010 ▶), Wriedt *et al.* (2009) ▶; Boeckmann *et al.* (2010 ▶). For a related structure, see: Manson *et al.* (2003 ▶). For a description of the Cambridge Structural Database, see: Allen (2002 ▶).
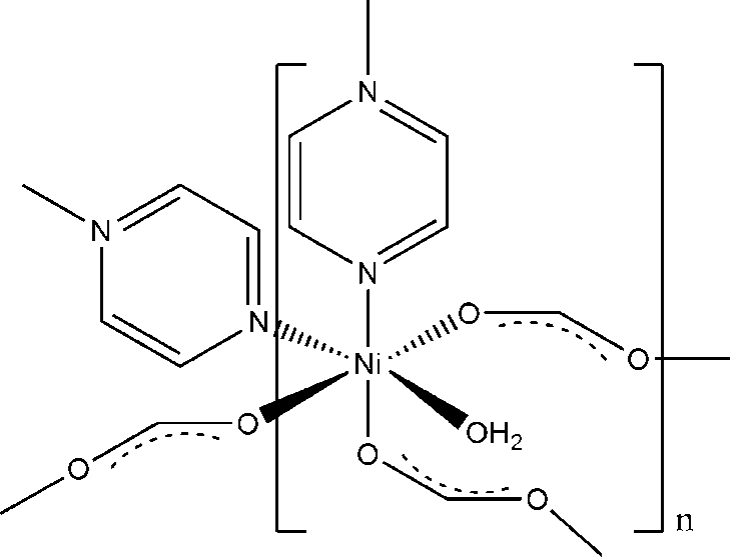


## Experimental

### 

#### Crystal data

[Ni(CHO_2_)_2_(C_4_H_4_N_2_)(H_2_O)]
*M* *_r_* = 246.85
Monoclinic, 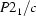

*a* = 7.8169 (4) Å
*b* = 7.0077 (3) Å
*c* = 15.6586 (7) Å
β = 98.971 (4)°
*V* = 847.26 (7) Å^3^
*Z* = 4
Mo *K*α radiation
μ = 2.29 mm^−1^
*T* = 293 K
0.19 × 0.15 × 0.12 mm

#### Data collection

Stoe IPDS-2 diffractometer
Absorption correction: numerical (*X-SHAPE* and *X-RED32*; Stoe, 2008 ▶) *T*_min_ = 0.658, *T*_max_ = 0.770
15596 measured reflections
2291 independent reflections
2091 reflections with *I* > 2σ(*I*)
*R*_int_ = 0.036

#### Refinement

*R*[*F* ^2^ > 2σ(*F* ^2^)] = 0.025
*wR*(*F* ^2^) = 0.059
*S* = 1.10
2291 reflections
128 parameters
H-atom parameters constrained
Δρ_max_ = 0.47 e Å^−3^
Δρ_min_ = −0.30 e Å^−3^

### 

Data collection: *X-AREA* (Stoe, 2008 ▶); cell refinement: *X-AREA*; data reduction: *X-AREA*; program(s) used to solve structure: *SHELXS97* (Sheldrick, 2008 ▶); program(s) used to refine structure: *SHELXL97* (Sheldrick, 2008 ▶); molecular graphics: *XP* in *SHELXTL* (Sheldrick, 2008 ▶); software used to prepare material for publication: *XP* in *SHELXTL* and *DIAMOND* (Brandenburg, 2011 ▶).

## Supplementary Material

Crystal structure: contains datablocks I, global. DOI: 10.1107/S1600536811011913/bt5501sup1.cif

Structure factors: contains datablocks I. DOI: 10.1107/S1600536811011913/bt5501Isup2.hkl

Additional supplementary materials:  crystallographic information; 3D view; checkCIF report

## Figures and Tables

**Fig. 1.**
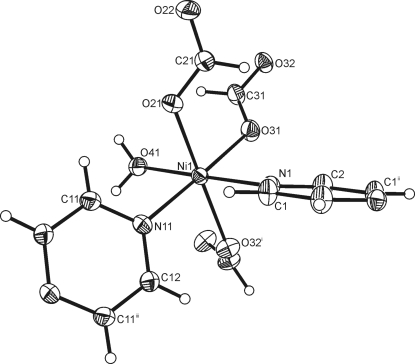
: Crystal structure of the title compound with labelling and displacement ellipsoids drawn at the 50 % probability level. Symmetry codes: i = -x+1, y+1/2 i = -x+1,y+1/2,-z+3/2; ii = -x+1,-y,-z+1; iii = -x,-y+1,-z+1.

**Fig. 2.**
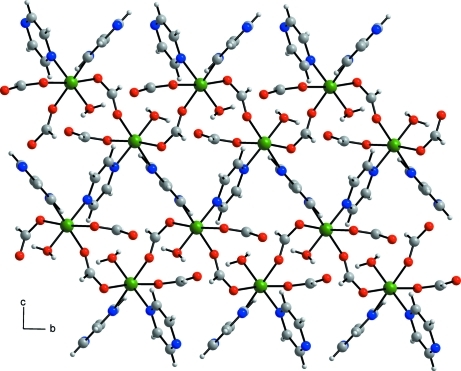
: Crystal structure of the title compound with view along the crystallographic a-axis.

## supplementary crystallographic information

### Comment

In our recent work on the synthesis, structures and properties of new coordination polymers based on paramagnetic transition metal, small-sized anions and *N*-donor ligands, we have shown that new ligand-deficient coordination polymers based on transition metal thiocyanates and selenocyanates can be prepared by thermal decomposition reactions (Wriedt, Jess & Näther, 2009 and Boeckmann & Näther, 2010). Later we have shown that also metal formates can be prepared by this route (Boeckmann, Wriedt & Näther, 2010). Within this project we tried to prepare new ligand-rich precursor compounds based on nickel(II) formate and pyrazine which resulted in the formation of the title compound that were identified by single crystal X-ray diffraction.

In the crystal structure of the title compound, each nickel(II) cation is coordinated by three bridging formato anions, two bridging pyrazine ligands and one water molecule (Fig. 1). The NiO_4_N_2_ octahedron is slightly distorted with Ni—OCHO distances between 2.0378 (11) Å and 2.0643 (12) Å and one Ni—OH_2_ distance of 2.0420 (11) Å as well as two long Ni—N distances of 2.1066 (13) Å and 2.1171 (12) Å. The angles around the metal atoms range from 84.14 (5)° to 95.94 (5)° and from 173.18 (5)°  to 179.19 (5)°. The nickel(II) cations are connected via *µ*-1,3 bridging formato anions into two dimensional Ni(O_2_CHO)_2_ layers that are further linked by the pyrazine ligands into a 3D coordination network (Fig. 2). The Ni—Ni distances between next neighboured Ni atoms ranges from 6.9770 (4) Å to 7.0689 (4) Å.

It must be noted that according to a search in the CCDC database (ConQuest Ver.1.12.) (Allen, 2002) compounds based on nickel(II) formate and pyrazine are unknown but with copper(II) formate one strcuture is reported (Manson *et al.*, 2003).

### Experimental

Nickel formate dihydrate [Ni(CHO_2_)_2_.H_2_O] and pyrazine were obtained from Alfa Aesar. All chemicals were used without further purification. 0.25 mmol (46 mg) Ni(CHO_2_)_2_.H_2_O and 0.5 mmol (40 mg) pyrazine were reacted in 2 ml water. Light blue block-shaped single crystals of the title compound were obtained after a few days at room temperature.

### Refinement

The C-H H atoms were positioned with idealized geometry and were refined isotropic with *U*_iso_(H) = 1.2*U*_eq_(C) and C—H distances of 0.93 Å using a riding model. The O-H H atoms were located in difference map, their bond lengths were set to 0.82 Å and afterwards they were refined isotropic with *U*_iso_(H) = 1.5*U*_eq_(O) using a riding model.

### Crystal data

**Table:**

[Ni(CHO_2_)_2_(C_4_H_4_N_2_)(H_2_O)]	*F*(000) = 504
*M**_r_* = 246.85	*D*_x_ = 1.935 Mg m^−^^3^
Monoclinic, *P*2_1_/*c*	Mo *K*α radiation, λ = 0.71073 Å
Hall symbol: -P 2ybc	Cell parameters from 15596 reflections
*a* = 7.8169 (4) Å	θ = 2.6–29.3°
*b* = 7.0077 (3) Å	µ = 2.29 mm^−^^1^
*c* = 15.6586 (7) Å	*T* = 293 K
β = 98.971 (4)°	Block, light blue
*V* = 847.26 (7) Å^3^	0.19 × 0.15 × 0.12 mm
*Z* = 4	

### Data collection

**Table:**

Stoe IPDS-2 diffractometer	2291 independent reflections
Radiation source: fine-focus sealed tube	2091 reflections with *I* > 2σ(*I*)
graphite	*R*_int_ = 0.036
ω scan	θ_max_ = 29.3°, θ_min_ = 2.6°
Absorption correction: numerical (*X-SHAPE* and *X-RED32*; Stoe, 2008)	*h* = −10→10
*T*_min_ = 0.658, *T*_max_ = 0.770	*k* = −9→9
15596 measured reflections	*l* = −21→21

### Refinement

**Table:**

Refinement on *F*^2^	Secondary atom site location: difference Fourier map
Least-squares matrix: full	Hydrogen site location: inferred from neighbouring sites
*R*[*F*^2^ > 2σ(*F*^2^)] = 0.025	H-atom parameters constrained
*wR*(*F*^2^) = 0.059	*w* = 1/[σ^2^(*F*_o_^2^) + (0.025*P*)^2^ + 0.4622*P*] where *P* = (*F*_o_^2^ + 2*F*_c_^2^)/3
*S* = 1.10	(Δ/σ)_max_ = 0.001
2291 reflections	Δρ_max_ = 0.47 e Å^−^^3^
128 parameters	Δρ_min_ = −0.30 e Å^−^^3^
0 restraints	Extinction correction: *SHELXL*, Fc^*^=kFc[1+0.001xFc^2^λ^3^/sin(2θ)]^-1/4^
Primary atom site location: structure-invariant direct methods	Extinction coefficient: 0.0158 (13)

### Special details

**Table:**

Geometry. All esds (except the esd in the dihedral angle between two l.s. planes) are estimated using the full covariance matrix. The cell esds are taken into account individually in the estimation of esds in distances, angles and torsion angles; correlations between esds in cell parameters are only used when they are defined by crystal symmetry. An approximate (isotropic) treatment of cell esds is used for estimating esds involving l.s. planes.
Refinement. Refinement of F^2^ against ALL reflections. The weighted R-factor wR and goodness of fit S are based on F^2^, conventional R-factors R are based on F, with F set to zero for negative F^2^. The threshold expression of F^2^ > 2sigma(F^2^) is used only for calculating R-factors(gt) etc. and is not relevant to the choice of reflections for refinement. R-factors based on F^2^ are statistically about twice as large as those based on F, and R- factors based on ALL data will be even larger.

### Fractional atomic coordinates and isotropic or equivalent isotropic displacement parameters (Å^2^)

**Table:**

	*x*	*y*	*z*	*U*_iso_*/*U*_eq_	
Ni1	0.30005 (2)	0.25657 (3)	0.649349 (11)	0.01731 (8)	
N1	0.41659 (17)	0.10169 (18)	0.55756 (8)	0.0205 (2)	
C1	0.3337 (2)	0.0453 (2)	0.48084 (10)	0.0250 (3)	
H1	0.2169	0.0747	0.4654	0.030*	
C2	0.5831 (2)	0.0558 (2)	0.57642 (10)	0.0257 (3)	
H2	0.6452	0.0925	0.6294	0.031*	
N11	0.12229 (17)	0.39364 (18)	0.55394 (8)	0.0209 (2)	
C11	−0.0463 (2)	0.3831 (2)	0.55924 (11)	0.0241 (3)	
H11	−0.0827	0.3027	0.6001	0.029*	
C12	0.1684 (2)	0.5112 (2)	0.49450 (10)	0.0234 (3)	
H12	0.2847	0.5224	0.4891	0.028*	
O21	0.11734 (15)	0.04433 (16)	0.65066 (8)	0.0275 (3)	
O22	0.0548 (2)	−0.25397 (17)	0.67969 (12)	0.0434 (4)	
C21	0.1545 (2)	−0.1280 (2)	0.66185 (12)	0.0279 (3)	
H21	0.2693	−0.1604	0.6602	0.042*	
O31	0.46637 (15)	0.11818 (18)	0.74213 (8)	0.0282 (3)	
O32	0.51992 (15)	−0.03704 (17)	0.86756 (7)	0.0260 (2)	
C31	0.4364 (2)	0.0746 (2)	0.81445 (11)	0.0258 (3)	
H31	0.3479	0.1347	0.8375	0.039*	
O41	0.18980 (15)	0.40396 (16)	0.73925 (7)	0.0241 (2)	
H1O	0.1126	0.3547	0.7617	0.036*	
H2O	0.1500	0.5093	0.7246	0.036*	

### Atomic displacement parameters (Å^2^)

**Table:**

	*U*^11^	*U*^22^	*U*^33^	*U*^12^	*U*^13^	*U*^23^
Ni1	0.01857 (11)	0.01683 (11)	0.01653 (11)	0.00177 (7)	0.00272 (7)	0.00092 (7)
N1	0.0220 (6)	0.0203 (6)	0.0198 (6)	0.0012 (5)	0.0049 (5)	−0.0023 (5)
C1	0.0200 (7)	0.0306 (8)	0.0238 (7)	0.0045 (6)	0.0020 (6)	−0.0043 (6)
C2	0.0230 (7)	0.0321 (8)	0.0212 (7)	0.0019 (6)	0.0007 (6)	−0.0070 (6)
N11	0.0214 (6)	0.0199 (6)	0.0206 (6)	0.0019 (5)	0.0007 (5)	0.0018 (5)
C11	0.0233 (7)	0.0245 (7)	0.0243 (7)	0.0000 (6)	0.0030 (6)	0.0059 (6)
C12	0.0194 (7)	0.0258 (7)	0.0248 (7)	0.0013 (6)	0.0029 (6)	0.0035 (6)
O21	0.0261 (6)	0.0184 (5)	0.0390 (7)	−0.0001 (4)	0.0077 (5)	0.0045 (5)
O22	0.0477 (8)	0.0184 (6)	0.0720 (11)	0.0009 (5)	0.0340 (8)	0.0041 (6)
C21	0.0283 (8)	0.0206 (7)	0.0372 (9)	0.0027 (6)	0.0128 (7)	0.0011 (6)
O31	0.0273 (6)	0.0356 (6)	0.0218 (5)	0.0097 (5)	0.0043 (5)	0.0080 (5)
O32	0.0296 (6)	0.0276 (6)	0.0208 (5)	0.0089 (5)	0.0039 (4)	0.0040 (4)
C31	0.0286 (8)	0.0266 (8)	0.0227 (7)	0.0087 (6)	0.0054 (6)	0.0020 (6)
O41	0.0264 (6)	0.0208 (5)	0.0271 (6)	0.0015 (4)	0.0101 (4)	−0.0002 (4)

### Geometric parameters (Å, °)

**Table:**

Ni1—O31	2.0378 (11)	C11—C12^iii^	1.385 (2)
Ni1—O41	2.0420 (11)	C11—H11	0.9300
Ni1—O32^i^	2.0636 (11)	C12—C11^iii^	1.385 (2)
Ni1—O21	2.0643 (12)	C12—H12	0.9300
Ni1—N11	2.1066 (13)	O21—C21	1.2479 (19)
Ni1—N1	2.1171 (12)	O22—C21	1.238 (2)
N1—C2	1.328 (2)	C21—H21	0.9300
N1—C1	1.333 (2)	O31—C31	1.230 (2)
C1—C2^ii^	1.382 (2)	O32—C31	1.2492 (19)
C1—H1	0.9300	O32—Ni1^iv^	2.0636 (11)
C2—C1^ii^	1.382 (2)	C31—H31	0.9299
C2—H2	0.9300	O41—H1O	0.8200
N11—C12	1.334 (2)	O41—H2O	0.8200
N11—C11	1.335 (2)		
			
O31—Ni1—O41	92.30 (5)	C1^ii^—C2—H2	119.2
O31—Ni1—O32^i^	93.04 (5)	C12—N11—C11	117.00 (13)
O41—Ni1—O32^i^	95.94 (5)	C12—N11—Ni1	123.80 (11)
O31—Ni1—O21	90.89 (5)	C11—N11—Ni1	118.62 (10)
O41—Ni1—O21	89.46 (5)	N11—C11—C12^iii^	121.75 (14)
O32^i^—Ni1—O21	173.18 (5)	N11—C11—H11	119.1
O31—Ni1—N11	178.28 (5)	C12^iii^—C11—H11	119.1
O41—Ni1—N11	87.46 (5)	N11—C12—C11^iii^	121.25 (14)
O32^i^—Ni1—N11	88.68 (5)	N11—C12—H12	119.4
O21—Ni1—N11	87.40 (5)	C11^iii^—C12—H12	119.4
O31—Ni1—N1	86.89 (5)	C21—O21—Ni1	123.54 (11)
O41—Ni1—N1	179.19 (5)	O22—C21—O21	125.53 (16)
O32^i^—Ni1—N1	84.14 (5)	O22—C21—H21	118.4
O21—Ni1—N1	90.52 (5)	O21—C21—H21	115.9
N11—Ni1—N1	93.35 (5)	C31—O31—Ni1	125.64 (11)
C2—N1—C1	116.76 (13)	C31—O32—Ni1^iv^	130.50 (10)
C2—N1—Ni1	118.93 (11)	O31—C31—O32	127.86 (15)
C1—N1—Ni1	124.31 (10)	O31—C31—H31	120.4
N1—C1—C2^ii^	121.65 (14)	O32—C31—H31	111.6
N1—C1—H1	119.2	Ni1—O41—H1O	120.0
C2^ii^—C1—H1	119.2	Ni1—O41—H2O	116.4
N1—C2—C1^ii^	121.59 (15)	H1O—O41—H2O	103.1
N1—C2—H2	119.2		

Symmetry codes: (i) −*x*+1, *y*+1/2, −*z*+3/2; (ii) −*x*+1, −*y*, −*z*+1; (iii) −*x*, −*y*+1, −*z*+1; (iv) −*x*+1, *y*−1/2, −*z*+3/2.
